# Stress Response and Cognitive Performance Modulation in Classroom versus Natural Environments: A Quasi-Experimental Pilot Study with Children

**DOI:** 10.3390/ijerph15061098

**Published:** 2018-05-28

**Authors:** Lærke Mygind, Matt P. Stevenson, Lasse S. Liebst, Ivana Konvalinka, Peter Bentsen

**Affiliations:** 1 Health Promotion, Steno Diabetes Center Copenhagen, The Capital Region of Denmark, Niels Steensens Vej 6, DK-2820 Gentofte, Denmark; peter.bocz.bentsen@regionh.dk; 2 Department of Geosciences and Natural Resource Management, University of Copenhagen, Nødebovej 77A, DK-3480 Fredensborg, Denmark; mps@ign.ku.dk; 3 Department of Sociology, University of Copenhagen, Øster Farimagsgade 5, DK-1014 Copenhagen, Denmark; lsl@soc.ku.dk; 4 Section for Cognitive Systems, Department of Applied Mathematics and Computer Science, Technical University of Denmark, Richard Petersens Plads, 2800 Kgs. Lyngby, Denmark; ivako@dtu.dk

**Keywords:** affect, autonomic nervous system, concentration, green space, education outside the classroom

## Abstract

Stress during childhood can have mental and somatic health influences that track throughout life. Previous research attributes stress-reducing effects to natural environments, but has mainly focused on adults and often following leisurely relaxation in natural environments. This pilot study explores the impact of natural environments on stress response during rest and mental load and cognitive performance in 47 children aged 10–12 years in a school context. Heart rate variability measures indexing tonic, event, and phasic vagal tone and attention scores were compared across classroom and natural environments. Tonic vagal tone was higher in the natural environment than the classrooms, but no differences were found in event or phasic vagal tone or cognitive performance measures. These findings suggest a situational aspect of the conditions under which natural environments may give rise to stress-buffering influences. Further research is warranted to understand the potential benefits in a real-life context, in particular with respect to the underpinning mechanisms and effects of accumulated exposure over time in settings where children spend large proportions of time in natural environments.

## 1. Introduction

The World Health Organization estimated that more than half the global population in 2015 was living in urban environments, with an expected growth rate of approximately 1.84% per year between 2015 and 2020 [1]. Urban lifestyles have been found to encompass a set of adverse psychosocial and environmental influences that facilitate chronic stress [2]. In line with this, current urban living was observed to be associated with increased amygdala activity, indicative of stress experience, and urban upbringing with poorer social stress processing at a neural level [3]. Similarly, a recent study found that remoteness to forests and urban green space amongst urban dwellers was associated with impaired amygdala integrity—a neural marker of stress coping [4]. Altered stress response related to urban environments may play an indirect role in the development or maintenance of mental health problems. For example, schizophrenia rates were found to be up to 56% higher when comparing most to least urbanized regions [5,6]. Likewise, Haddad and co-authors [7] found a strong correlation between early-life urbanity and neural markers of psychosocial stress that have been linked to an increased risk of schizophrenia.

There is convincing evidence that early-life stress can influence mental and cognitive health and the individual’s response to stressors throughout their lifetime [8]. Additionally, there are indications that early-life stress may promote a neurobiological susceptibility to various stress-related illnesses later in life [9]. Toxic stress during childhood may occur as a response to traumatizing events, such as neglect or sexual abuse, but also from a cumulative exposure to psychosocial and environmental stressors that can lead to a pathogenic overload of the neurobiological stress-system [10]. Consequently, it is imperative to develop and evaluate early childhood initiatives to reduce psychosocial and environmental influences that cumulatively may facilitate toxic stress. In this study, we investigate whether exposure to natural environments in a school setting is associated with reduced stress and improved cognitive performance in comparison to classroom environments.

### 1.1. Natural Learning Environments to Reduce Early-Life Stress

A growing body of research has shown that living close to natural landscapes is associated with enhanced social and mental health [11], wellbeing, mood, cognition [12], amygdala integrity [4], and hard-end indicators such as longevity [13] and mortality [14]. In a longitudinal study, Alcock, White, Wheeler, Fleming, and Depledge [15] provided evidence that green areas were beneficial for people’s mental health. Moreover, Roe et al. [16] found that more green space in socially disadvantaged areas was associated with lower levels of perceived stress and psychophysiological stress as measured by cortisol secretion. In addition to this bulk of mainly correlational research, a growing number of small-scale experimental studies showed positive effects of seated relaxation and slow-paced walking in forest environments on cortisol, pulse rate, blood pressure, parasympathetic, and sympathetic nerve activity [17]. While there is evidence that contact with nature is also beneficial for children’s general wellbeing and cognitive function [18,19,20], children today are spending less time in nature compared to previous generations [21,22] and do not have equal access to natural environments [23]. Since children spend most of their waking hours in school [24], and there is growing recognition that the institutions in which children live and develop are highly influential on children’s short and long-term wellbeing and development [10], it is worth considering how the school setting could accommodate these new trends.

While most child-oriented research has utilized parental reports and is acknowledged to be susceptible to bias [19], recent small-scale studies utilized psychophysiological measures to index stress. Berto and Barbiero [18] observed that the heart rate (HR) and blood pressure of middle school-aged children was lower after a 90-min woodland walking session than after 90 min of free play in the school playground. However, a classroom-based mindful silence exercise provided similar results to the woodland exposure. HR and blood pressure are influenced by psychosocial stress, but also other circulatory and respiratory systems, thereby not providing strong psychophysiological indexes. In a prospective longitudinal study, Dettweiler, Becker, Auestad, Simon, and Kirsch [25] found that children who participated in teaching in a natural environment displayed a larger decline in salivary cortisol levels over a school day than children who went to school as normal, irrespective of time of the year and levels of physical activity. Since individual school subjects had not been absolutely synchronized, a bias was possible due to differences in mental load [25].

### 1.2. The Present Study

#### 1.2.1. Situational Autonomic Indexes of Stress

In this study, the children were exposed to the same mental load in the natural and classroom environments to isolate the effects of the environments on psychosocial stress indexed by so-called tonic and phasic vagal tone. Tonic cardiac vagal tone is considered to indicate the modulation of the vagus nerve, that is, the contribution of the parasympathetic nervous system, on the HR during sitting or supine rest [26,27]. Phasic vagal tone indexes the vagal withdrawal that occurs during the transition from rest to an event [28]. Higher levels of tonic vagal tone indicates a larger resource of adaptability to external events, and has been linked to improved emotion recognition [29], and inversely associated with anxiety disorders [30], depression [31], and alcohol dependence [32], a behavior commonly associated with poor self-regulation. Heart rate variability, a peripheral indicator of vagal tone, is associated with age and gender, with males tending to display larger levels than females [33], and has shown to increase until approximately the age of ten after which the development levels out [34,35].

#### 1.2.2. Neurobiological Connections between Stress and Attention

While it may not be the primary function of schools to improve children’s wellbeing and bolster against cumulative stress during early life [36], there is evidence that the emotional climate of classrooms and schools has an impact on academic outcomes [37] and that stress experience may impede the neurobiological systems involved with learning. Previous neuroscientific research indicated that stress exposure activates subcortical regions of the brain, such as the amygdala, leading to vagal withdrawal and disruption of connections in phylogenetically more recent neural systems [38,39]. This includes the prefrontal cortex, a region that includes the neural circuitry underlying directed attention and other executive functions [40,41]. Across the body of neurobiological research, Arnsten [39] found that severe stress exposure switched the brain into a fight-or-flight mode that under given circumstances has survival value, while weakening higher-order top-down processes. Although the main focus was on severe acute stress or chronic stress, mild acute stress also affected these neural mechanisms, although to a lesser degree [39]. Indicatively, several studies have linked tonic vagal tone with cognitive performance [27,42,43,44,45]. Furthermore, a moderate, vagal withdrawal as a response to a mentally demanding task was found to be optimal for attention performance amongst three to five year olds [46].

#### 1.2.3. Aims of the Present Study

The primary purpose of this pilot study was to investigate the differential effects of natural environments compared to classroom environments on the psychophysiological stress system, indicated by vagal tone, in a school context. Further, we investigated whether an effect of the natural environment is also observed in terms of the children’s cognitive performance, and whether vagal tone mediates this effect.

## 2. Materials and Methods

We applied a quasi-experimental, within-subjects design in which the participating children performed the same activities and cognitive tasks to retain a similar mental load across the two types of environment. Sequence order was counter-balanced to reduce order effects. The data were generated between April and June 2016.

### 2.1. Sampling and Population

**Figure 1:**
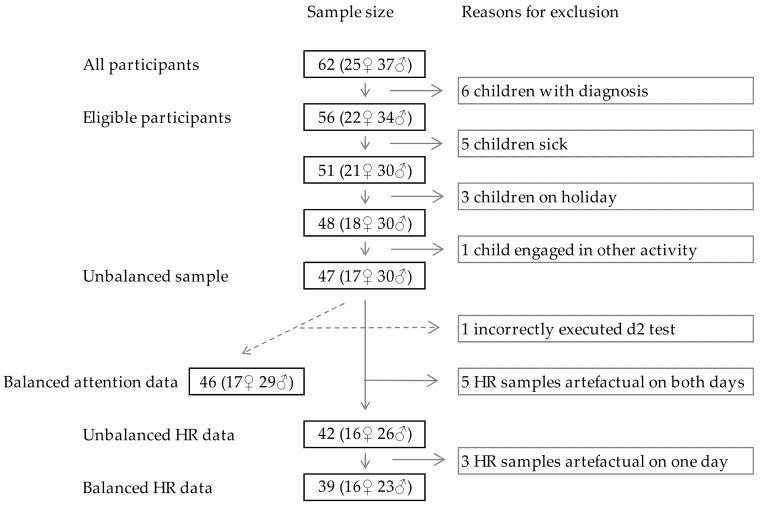
Reasons for participant exclusion. The unbalanced sample indicates that data includes missing observations, for example missing attention scores from one participant on one of the measurement days. The balanced attention scores and heart rate (HR) data includes no missing observations. The artefactual HR data samples are excluded due to many changes in beat-to-beat rhythm that are very different from the individuals’ normal beat-to-beat rhythm.

To avoid reactivity to the new situation, that is, the novelty effect [47], our population consisted of typically developing children who were accustomed to education in natural environments. Four schools that were part of the Danish ministerial education outside the classroom (EOtC) [48,49] development project “*Development of Udeskole*” [50], and located in the vicinity of natural environments, were invited to participate in the study. On the basis of these criteria, these schools were expected to have teachers and classes regularly performing EOtC in the nearby natural environments. Two schools responded positively, one negatively, and one did not reply. Both participating schools were located in affluent areas north of Copenhagen. School A covered grades zero to five (ages five to 12), with 245 students, and school B covered all elementary school grades, zero to nine (ages five to 16), with 699 students, in the 2015/2016 school year. Two fifth-grade classes from school A and one fourth-grade class from school B participated. Since mental disorders have been associated with irregularities in vagal tone measures [51], five children were excluded from the study on the basis of being diagnosed with ADHD and one child with autism. Fifty-six fourth- and fifth-grade students (10–12 years), who had no diagnoses of mental disorders, participated. Informed consent was gathered from the children and their parents prior to data generation. The study was conducted in accordance with the Declaration of Helsinki, and the protocol was exempt from ethical review by the Danish Data Protection Agency. The reasons for exclusion are listed in Figure 1.

### 2.2. Learning Environments

The classroom environments utilized in School A were similar: the spaces were airy and colored in mainly white and gray tones with high-quality furniture, fixtures, equipment, and views of green surroundings. One classroom had a few plants, the other did not. All children from School A went to the same forested area, where activities were performed at the top of a grassy slope surrounded by trees on three perimeters overlooking a lake. Only a single permanent bench was available, so the children brought yoga mats on both occasions. It was windy and chilly during the visit to the natural environment for the children of class A1 and warmer and clear for those of class A2. The School B classroom was darker, more crowded, noisier, and had no plants and limited view to greenery. Furthermore, the classroom had no ventilation or air conditioning, which was emphasized by the hot weather on the day of measurement. On the day of measurement in the natural environment, the weather was sunny and the ambient temperature was comfortable. The natural environment was shaded and sheltered by trees encircling the benches that were located there permanently.

### 2.3. Measures and Instruments

#### 2.3.1. Stress Response

Polar Team2 Pro (Polar Electro Oy, Kempele, Finland) chest-strapped HR monitors were used to record the children’s inter-beat R-R intervals with millisecond accuracy. The mobile devices for the measurement of R-R intervals by Polar generally show excellent agreement with measurements obtained from electrocardiography (ECG) for both adults [52,53,54] and children [55] (For recent empirical studies in which the Polar Team2 Pro system was applied to dynamically explore heart rate variability (HRV) in different contexts, see Noah et al. [56] and Silva-Urra et al. [57]).

Recordings were generated during five minutes of supine rest and the attention task, for a total of four minutes and 40 seconds. Raw R-R intervals were exported from the Polar Team2 software to Microsoft Excel, where a manual data check of the epochs of interest was performed. Artefacts and ectopic beats in the data were interpolated by replacing the artefactual R-R interval with the average of three neighboring R-R intervals. If artefactual R-R intervals made up more than 20% of the selected data segment, the data were considered of poor quality and eliminated. Data processing and analysis were performed with the free professional HRV analysis software of the University of Kuopio, Finland [58]. Following the study of Michels et al. [59], who also used a Polar device for HRV analysis with children, the R-R intervals were detrended using a smoothness priors algorithm [58], with alpha set at 300 and cubic interpolation was carried out to replace missing heart beats at the default rate of 4 Hz.

Root mean square of successive differences (RMSSD) was calculated from the data during supine rest, to indicate tonic cardiac vagal tone (labelled as TONIC in the Supplementary Data File), and during execution of the d2 test, to indicate event vagal tone (labelled as EVENT). The difference between tonic and event vagal tone was calculated to index phasic vagal tone (labelled as PHASIC).

#### 2.3.2. Cognitive Performance

Cognitive performance was measured by means of the d2 Test [60]. The d2 Test is a paper and pencil letter cancellation test in which the letter *d* with two apostrophes must be identified among a number of distractors. Letters are arranged in 14 rows, which the children are given 20 s to finish and then move on to the next line.

In this study, the d2 Test was utilized as comparable mental load across the two environments, but also to provide a measure of cognitive performance. Three parameters were utilized to quantify the speed with which stimuli were processed and the accuracy of the performance: (1) the total number of symbols processed (TN, also labelled TN in Supplementary Data File); (2) the total number of errors, including erroneously marked distractors and omitted stimuli (E, also labelled E in Supplementary Data File); and (3) the total number of symbols processed minus the erroneously marked distractors (TN-E, also labelled as TN-E in Supplementary Data File). The construct validity of the latter index, TN-E, to reflect complex attention, in particular, complex scanning, visual tracking, and sustained attention, is well-established [61]. Furthermore, the internal reliability of all parameters have been found to be high (0.9–0.95) [60].

### 2.4. Procedure

The HR monitors, an illustration of the placement of these devices, and the procedure were presented to the children at the beginning of the first day of measurement. Children were instructed to abstain from physical activity on both days of measurements, and food and caffeinated drinks for the last two hours before the measurements. After a general introduction, the children went individually to a quiet room where trained research assistants helped them place the monitor accurately. The children wore the devices throughout both days.

Sequence order was determined by the weather conditions and could not be randomized. Consequently, one class was first exposed to the natural environment and then the classroom environment. The other two classes were first exposed to the classroom environment and then the natural environment.

Once all the children were fitted with their monitors, they sat reading quietly in either the classroom or the natural environment for approximately an hour. Subsequently, tonic vagal tone was measured during supine rest. Since posture is known to influence autonomic outflow [51], the children performed a five-minute low-demand cognitive exercise (a simple letter cancellation task) to allow for an adaptation to the sitting position before performing the d2 Test. The low-demand, adaptation exercise was followed by a short break (approximately one minute), after which the children executed the d2 Test. The entire session, that is, including instructions, supine measurements, low-demand, adaptation exercise, short break and the d2 Test, took approximately 20 min.

On the second day of measurement, a week after the first session, the children went through the same procedure. The measurements were conducted at the same time of the day on both measurement days. At the end of each day, the monitors were handed back to the researchers.

### 2.5. Statistical Analysis

After data management and reduction, the data were exported to SPSS v.25 (IBM, New York, NY, USA). Preliminary analyses were performed on the primary measures, including exploration of the data for normality and outliers. The main measures included tonic, event, and phasic vagal tone, and three cognitive performance parameters: TN-E, TN and E.

For each primary measure an estimate of the fixed effect of the individual environments was calculated utilizing generalized estimating equations (GEE), using the GENLIN procedure in SPSS [62]. A major advantage of GEE is that it handles correlated and clustered data, is robust to covariance misspecification, and adequately handles missing data [63]. The GEE algorithm is similar to random or mixed effects models, but does not rely on specification of the structure of random effects to disentangle these from residuals and thereby absorbs non-independence in the residuals in a very flexible way.

Psychophysiological variable residuals were not normally distributed, as indicated by histograms, P-P plots, and the Shapiro-Wilk test (statistics not reported here). To meet the assumption of normality of response variables in GEE, psychophysiological measures were log-transformed (labelled logTONIC, logEVENT, and logPHASIC in Supplementary Data File). Subject ID (labelled ID) was specified as within-subjects variable with an unstructured covariance matrix. The environments (labelled ENVIRONMENT: 1 = natural environment and 2 = classroom environment) were set as factor and condition sequence (labelled SEQUENCE: 1 = first day of measurement and 2 = second day of measurement), age and sex (labelled age and sex: 0 = female and 1 = male) as covariates. The effect of the environments was of primary interest whereas the other variables were included in order to reduce residual variability. Estimated marginal means (EMMs) were calculated to illustrate differences in adjusted means.

## 3. Results

### 3.1. Observed Distributions on Primary Measures

Of the 47 participating children, 18 were girls and the mean age was 10.9 years (SD: 0.71). One class of 14 children was first exposed to the natural environment and two classes with a total of 34 children had their first day of measurement in the classrooms.

Table 1 summarizes the scores from the observed means and medians of the primary measures in the two environments. Since the psychophysiological variables did not follow a normal distribution, they are described according to their median and interquartile range while means are used to describe TN-E, TN and E.

### 3.2. Stress Response in the Natural and Classroom Environments

The GEE indicated that the environments predicted tonic vagal tone (β = 1.13 ± 1.06, *p* = 0.031), but not event (β = 1.1 ± 1.07, *p* = 0.161) and phasic (β = −0.94 ± 1.07, *p* = 0.366) vagal tone while controlling for age, gender and condition sequence (see Table 2). The EMMs, that is, means that are estimated from the fitted model, indicated that the mean tonic vagal tone was higher in the natural environments (EMM = 71.38) than the classroom environments (EMM = 63.27). The boys generally had higher tonic (*p* = 0.013) and event vagal tone (*p* < 0.000) than the girls.

### 3.3. Cognitive Performance in the Natural and Classroom Environments

Neither TN-E (β = −1.74 ± 5.29, *p* = 0.691), TN (β = −2.1 ± 5.05, *p* = 0.677), nor E (β = −0.22 ± 1.67, *p* = 0.898) appeared to be predicted by the environment factor while controlling for age, gender and condition sequence, as well as random subject influences. Condition sequence appeared to predict all the variation (*p* < 0.000) for both TN-E and TN in favor of the second time the children performed the attention task. Since we did not identify an effect of the natural environment on the attention scores, we did not perform a mediation analysis.

## 4. Discussion

The higher levels of tonic vagal tone found in the natural environments support the conclusions by Dettweiler et al. [25] who found that cortisol levels, indicative of stress response, were improved during teaching in natural environments and not in the classroom. However, we found no support of an environment-related difference in event, that is, on-task vagal tone, or phasic vagal tone, that is, vagal withdrawal in response to mental load. Our results suggest that the acute effects of the natural environment on the autonomic systems involved with stress processing are situation-dependent: it is during rest, that is, during break time or mental pauses, in which the influence of the natural environments ensues. This provides an interesting, tentative nuance to previous knowledge about the acute effects of natural environments on psychophysiological indexes of stress response that have predominantly explored passive utilization of natural environments, that is, leisurely walks or seated observations of natural scenery [17,64]. As has been previously established elsewhere [33,34,35], the boys displayed higher levels of both tonic and event vagal tone than the girls, across both environments.

The contribution of rest periods or time off-task to explain environmental effects on cognitive performance seems less clear. In contrast to research that has found that children perform better on cognitive tasks after exposure to natural environments [20,65,66,67], we found no indications of superior cognitive performance in the natural environments compared to the classrooms. While most acute exposure studies used passive task-free environmental exposures [68], several studies reported improved cognitive performance when participants were exposed to natural stimuli during task performance (e.g., [69]). However, it was also shown that ‘micro-breaks’ of only 40-s, visual exposures to natural stimuli were sufficient to improve cognitive performance [70]. Therefore, future studies should attempt to describe situations under which natural environments influence cognitive performance, especially in relation to real-world settings, such as schools, by establishing whether rest periods are a necessity.

In a study by van den Berg, Koole, and van der Wulp [71], the d2 Test was used to examine the restorative effects of virtual nature. Here, participants were exposed to a four-minute video clip showing images of chickens being decapitated and other animals being brutally killed to induce an affective state of stress previous to measurements. The authors reported a marginal difference in TN-E and TN (approximately 20 points for both) post exposure to the conditions, but did not report any test scores. Hence, while the difference could have been statistically insignificant, it is possible that the introduction of a stress element allowed for the differential relaxation potentials of the conditions to renew attentional capacity in the participants. However, neither of the three before-mentioned studies involving children, who identified an environmental effect on the various cognitive measures, utilized pre-testing mental load to fatigue mental capacity [65,66,67].

Ohly et al. [68] hypothesized that the more challenging cognitive tests, such as tests that require working memory and manipulation of series of numbers as seen in, for example, the Backwards Digit Span test, may tap into an aspect of attention that is restored after exposure to natural environments. The Backwards Digit Span test was used, amongst other tasks, in Faber Taylor and Kuo [65] and Faber Taylor et al. [66]. With the d2 Test mainly involving orienting attention to stimulus while refraining from crossing out distractors under time pressure, executive functions may not have been challenged in the same way as is required when performing the Backwards Digit Span test. However, Ohly et al. [68] emphasized that the relation remains uncertain. Nevertheless, it could be speculated that the d2 Test, without previous mental fatigue or stress and following renewal of resources, does not tap into an aspect of attention likely to be affected by the restoration potential offered by natural environments.

### Strengths, Limitations and Future Perspectives

This study aimed to provide a tentative account of situational aspects of the impacts of the environment on stress and cognitive performance. We presented neurobiological evidence for an interconnectivity between stress and attention [39] to illustrate ways in which sensorial and cognitive perception of the environment provides a feedback mechanism to the neural circuitry involved with attention. As proposed in neuropsychological theory [26,38] this in turn affects the resources available to perceive and adapt to external events, for example, to direct and sustain attention when this is required. On this basis, we suggested a mediation model between stress response and cognitive performance. Since we found no differences in cognitive performance between the environments, potentially due to aspects discussed above, we did not find it meaningful to perform mediation analyses.

Although our analysis provided provisional evidence that presence in the two environments differentially modulated stress response, it remains uncertain what mechanisms caused this difference. Consequently, further attention to the mechanisms by which natural environments reduce stress and induce restorative states should be given. While evidence is accumulating for stress and cognitive benefits of natural environments, there is a scarcity of research addressing why, when, where, and for whom the effects occur—and whether the effects may be attributed indirectly or directly to nature. It is, for example, possible that simply being outside (not necessarily in nature) and having more space facilitated a more relaxing environment. Noteworthy contributions to understanding the mechanisms have been made exploring social aspects, for example, experiencing nature alone or with a friend [72,73], types of natural environments, for example, open versus more densely vegetated landscapes [74], situational aspects, for example, restorative or non-restorative usage of the natural environment [75], and psychosocial aspects, for example, levels of stress previous to exposure [73]. Future research could apply so-called Realistic Evaluation [76] to systematically assess the mechanisms involved with the relations between natural environments, stress and cognition.

A concern in the present study relates to potential unmeasured confounding effects from differential external factors, for example, temperature and humidity. Ambient temperature, for example, has been linked to HRV with indications that warmer temperatures may be related to higher ratios of sympathetic to parasympathetic nerve activity [77], although the index used, that is, so-called LF/HF ratio, has been subject to severe criticism, for example, in [78]. However, other time domain and power spectral measures of vagal tone have elsewhere been inversely linked to ambient temperatures [79,80], and colder temperatures to generally higher levels of spectral power and spectral measures of vagal tone [81]. While the ambient temperature under the various conditions were likely to influence the HRV measures, both colder and warmer temperatures were experienced in the natural environments, evening out potential impacts. Furthermore, due to the specification of a repeated factor with an unstructured covariance matrix, we sought to statistically filter out this type of influence. In relation to the children’s performance on the attention scores, the conditions may also have introduced some bias: through our GEE analyses, we were able to discern a practice effect. However, this remains an uncertain factor that should be measured in future studies outside controllable lab settings.

A strength of this study is the method used to access stress response. By using chest-strapped HR monitors, the method provides dynamic measures of stress response that do not depend on accurate recall and which bypass self-representation, social desirability, and social norms [82]. The method thus has advantages over observation-, survey-, and interview-based methods often used in research about children and nature [19]. While other forms of direct and objective measurement of physiological stress response, such as infrared thermography, may offer a less obtrusive alternative, the strength of the chosen method lies in its potential to be used outside of controlled settings. There are certain benefits to using controlled, lab-like settings. It is, for example, possible to achieve a large degree of compliance, where the participants follow the instructions and the measurements are executed as planned, without disturbances from external sources. In these terms, the realities in which the measurements take place, such as within the structure of a school schedule or noise from other students, are external sources that complicate the measurement of the explored relation. However, these external influences are also fundamental to the actual realities in which the explored dynamic exists. In this sense, the method allows for an investigation of environmental effects on stress response and cognitive performance to be conducted within the boundaries of a school, in familiar physical surroundings and a social situation with which the children and teachers are accustomed. The method thereby allows for a larger ecological validity, which is central to the transferability of the conclusions, although perhaps at the cost of the greater effect sizes achieved in a lab-like setting. However, this study did not explore a typical school day and further research is warranted for the findings to be directly applicable to teaching activities in natural and classroom environments.

Given the relatively small sample size utilized in this pilot study, future studies are required to substantiate the understanding of the phenomenon. While the findings in the study are provisional, we wish to draw attention to the possible practical implications of this study for the organization of education and the challenges involved with urban lifestyles in which the majority of children grow up. The present study took place during quasi-experimental conditions in a school setting, where contact with nature had become routine. The participating children were recruited from schools that practiced EOtC and were positioned in close proximity to natural environments. As such, it is likely that the children also resided in proximity to natural environments. These children and schools are likely not representative of the general population and direct transfer of the findings to children who reside in urban areas or are not accustomed to EOtC may not be possible. It is unknown whether children residing in urban areas with larger exposure environmental stressors would be more or less susceptible to the potential benefits of nature when integrated into the school day. Therefore, there is a need for further studies including children who are not regularly exposed to EOtC in natural environments and schools that are not positioned in natural environments to ascertain potential benefits more widely. Additionally, while the main aim of the study was to explore dynamic aspects of stress response and cognitive performance, it remains unclear whether and how effects endure beyond the acute time and space, and during activities that are more similar to everyday school activities. Dettweiler et al. [25] provided the first study into the more long-term effects of nature-based EOtC on stress, although the results may be related to differences in subject content, that is, mental load. Genuine EOtC, where natural or other informal learning environments outside school buildings are used repeatedly for curricular learning activities, appears to be feasible nationwide, since this practice is already common and spreading throughout Denmark [50,83] and many other Western countries [84,85,86,87,88]. Therefore, using natural environments in educational activities could be a feasible intervention for reducing stress and thereby more widely enhancing wellbeing among children.

## 5. Conclusions

The aim of our study was to investigate whether exposure to natural environments in a school setting was associated with reduced stress and improved cognitive performance in comparison to classroom environments. Results indicated that tonic vagal tone, but not event or phasic vagal tone, was higher in the natural environments compared to the classroom environments. These findings suggest a situational aspect of the conditions under which natural environments may have stress buffering influences. In other words, potential stress buffering benefits may be accumulated during breaks and not cognitively demanding exercises. Boys exhibited lower levels of stress response than girls, across both environments. Although observed cognitive performance scores were relatively higher in the natural environment, GEE analysis showed that the difference was due to condition sequence, suggesting a practice effect. Since more children performed the cognitive task for the second time in the natural environment, the practice effect inflated observed cognitive scores in the natural environment. In other words, we could find no evidence to support that the children’s cognitive performances were improved in the natural environments compared to classrooms. This pilot study provided a tentative, quasi-experimental account of the stress buffering potential for utilizing natural environments in a school context. Further research is warranted to understand the potential benefits in a real-life context, in particular with respect to the underpinning mechanisms and effects of accumulated exposure over time in settings where children spend large proportions of time in natural environments.

## Figures and Tables

**Table 1:** Observed distributions of primary measures in the natural and classroom environments, split by sex.

	Natural		Classroom		
	Male	Female	Male	Female	*n*
Tonic vagal tone	100.74 (58.34–128.16)	57.45 (39.22–100.4)	83.15 (61.86–117.71)	53.94 (31.55–65.93)	41
Event vagal tone	52.55 (28.56–76.98)	28.46 (19.45–37.34)	42.95 (29.73–60.37)	29.7 (23.87–38.54)	41
Phasic vagal tone	54.92 (42.34–63.0)	52.28 (39.55–65.18)	49.92 (46.53–71.54)	57.6 (47.69–82.49)	39
TN-E	373.69 (82.53)	393.24 (99.42)	343.27 (67.28)	354.78 (77.98)	46
TN	388.45 (87.27)	405.0 (101.91)	357.43 (71.89)	365.11 (83.83)	46
E	14.76 (13.43)	11.76 (11.23)	14.17 (11.86)	10.33 (9.65)	46

Only balanced data, i.e., no missing observations, are included in the table. Observed distributions for psychophysiological measures are reported as median and interquartile range (in parenthesis) and for cognitive performance measures as mean and standard deviation (in parenthesis).

**Table 2:** Parameter estimates and estimated marginal means (EMMs) of the primary measures in the natural and classroom environments.

				N	C		
	β	SE	95% CI	EMM		*n*	*p*
Tonic vagal tone	1.13	1.06	1.01–1.26	71.38	63.27	41	0.031
Event vagal tone	1.1	1.07	0.96–1.25	40.27	36.74	42	0.161
Phasic vagal tone	−0.94	1.07	−1.07–0.82	53.28	56.66	40	0.366
TN-E	−1.74	5.29	−12.11–8.63	361.66	363.4	48	0.691
TN	−2.1	5.05	−11.99–1.79	374.5	376.6	48	0.677
E	−0.22	1.67	−3.5-3.07	12.98	13.18	48	0.898

SE, standard error, N, natural environment, C, classroom environment. Parameter estimates for psychophysiological measures were calculated from logarithmic values, but are in the table presented as the inverse function of the log value, i.e., in root mean square of successive differences (RMSSD).

## Supplementary Materials

The following is available online at http://www.mdpi.com/1660-4601/15/6/1098/s1, Supplementary Data File S1.
